# PALLIATIVE CARE FOR NURSING HOME RESIDENTS: APPLYING LESSONS LEARNED FROM COVID-19

**DOI:** 10.1093/geroni/igac059.741

**Published:** 2022-12-20

**Authors:** Kathleen Unroe

**Affiliations:** Indiana University Center for Aging Research, Regenstrief Institute, Inc., Indianapolis, Indiana, United States

## Abstract

Many people receive care near or at the end of life in nursing homes, including 70% of people with Alzheimer’s Disease and Related Dementias (ADRD). Studies have documented unmet needs for symptom management and frequent transitions of care for nursing home residents. Despite this, access to palliative care for nursing home residents is inconsistent. The COVID-19 pandemic both highlighted and exacerbated inequities in access to care, including in US-based nursing homes, as well as globally. COVID-19 specific guidance for nursing homes at state and federal levels, while designed to protect residents, contributed to increased social isolation and functional decline. Drawing upon data from an ongoing study to advance palliative care for residents living with ADRD, this presentation will highlight promising practices and opportunities to deliver palliative care in this setting.

